# Supplementary material for: User perspectives and preferences on a novel TB LAM diagnostic (Fujifilm SILVAMP TB LAM)–a qualitative study in Malawi and Zambia
Source: PLOS Glob Public Health. 2022 Jul 19;2(7):e0000672. doi: 10.1371/journal.pgph.0000672 (PMC10021253; doi:10.1371/journal.pgph.0000672)
Supplement: S1 File — A more detailed description of the current TB diagnostic algorithms in Malawi and Zambia is provided. In addition, we described clinical settings in both countries to allow a better understanding of the respective health care facilities where our study took place. (PDF) [file pgph.0000672.s001.pdf]

Supplement "User perspectives and preferences on a novel TB LAM diagnostic (Fujifilm SILVAMP TB LAM) – a qualitative study in Malawi and Zambia"

S1 File CONTEXT:

Current urine LAM guidelines and TB diagnosis algorithms in Malawi and Zambia in their respective health care settings

Malawi and Zambia suffer from a high TB and HIV burden [1, 2]. In Zambia, the HIV prevalence in adults is estimated to be around 11%, whereas approximately 15% of children suffer from HIV infection [3]. The TB/HIV co-infection rate is estimated to be between 50% and 70% [4]. In Malawi, 8.5% of adults and 0.7% of children were infected with HIV as of 2021 [5]. Of all HIV patients testing for TB, 63% tested positive leading to a high HIV/TB co-infection rate which underlines the importance of TB testing in HIV positive populations [6].

AlereLAM entered the market in 2013, and has been recommended by the World Health Organization (WHO) for TB diagnosis in PLHIV since 2015 [7]. However, uptake by national health systems has been slow, and as of 2019, only five countries had scaled up AlereLAM TB testing nationally [7, 8]. The recommended sputum-based TB test in Zambia and Malawi, Xpert MTB/RIF and Xpert Ultra MTB/RIF, is an automated molecular system that allows for rapid, simultaneous detection of both Mycobacterium tuberculosis (MTB) and resistance to rifampicin, a key first-line anti-TB drug [9], and its widely used for TB diagnostics across different countries. Xpert requires regular maintenance of the modules to execute TB and rifampicin resistance testing properly. This is part of the reason why Xpert machines are usually only available in (peri-)urban hospitals who fulfill requirements to install such device [10].

According to Malawi's HIV national guidelines from 2018, AlereLAM testing is recommended in routine testing for PLHIV with advanced HIV or severe illness regardless of the CD4 count (e.g. all admitted in-patients; patients with ≥30 breaths/min, a heart rate ≥120 beats/min; patients who are unable to walk unaided or who show ≥39°C). Moreover, PLHIV with a CD4 count bellow 200 cells/µ should receive LAM testing according to the HIV guidelines [11]. Slightly differentiating, the 2017 TB guidelines recommend AlereLAM for presumptive TB in PLHIV with advanced HIV or danger signs, only when Xpert is not available [12]. In Malawi, presumptive TB patients can receive testing at public primary health care facilities. However, means of Xpert testing are limited in rural primary health care facilities whereby at least one Xpert device is available per district [13]. In rural settings, TB testing is usually done by smear microscopy, however, collected sputum samples of high-risk patients are required to be sent to clinics offering Xpert testing [11]. As of 2021, 81 out of 392 TB diagnostic sites in Malawi offer TB testing through Xpert [14].

According to Zambia's HIV guidelines from 2020, AlereLAM testing is recommended in PLHIV with signs and symptoms of TB, or seriously ill PLHIV (with a CD4 count below 200 cells/µl for inpatients, or less than 100 cells/µl for outpatients) irrespective of signs and symptoms of TB, similar to the TB guidelines [4, 15]. In routine conditions, the required CD4 count in PLHIV is determined through a laboratory-based test from venous blood. As an alternative, Omega's Visitect CD4 test (henceforth called Visitect) is a POC test which uses finger prick blood and takes around an hour to give a result. CD4 testing was not conducted routinely in our study since the sites included patients with a known HIV diagnosis and therefore, not all interviewed healthcare professionals were familiar with performing these CD4 tests. However, some of the healthcare professionals interviewed for this study have worked with CD4 testing in other study settings.

In Zambia, Xpert testing is currently required for priority patients (e.g. PLHIV, children, EPTB, patients with risk of drug resistance). Samples of priority patients may be collected at all public hospitals including primary health care facilities but must be sent to facilities with an Xpert device to detect and identify TB and potential drug resistance. Non-priority patients may solely

receive diagnosis through smear microscopy [4]. Even though Xpert sites have tripled between 2016 and 2018 from 69 to 210 making Xpert available in all districts, modules are predominantly available in larger district hospitals [16].

1. Stop TB Partnership. Tuberculosis Situation in 2020 Zambia 2020 [29/03/2022]. Available from: https://www.stoptb.org/static_pages/ZMB_Dashboard.html.
2. Stop TB Partnership. Tuberculosis Situation in 2020 Malawi 2020 [29/03/2021]. Available from: https://www.stoptb.org/static_pages/MWI_Dashboard.html.
3. UNAIDS. Country progress report - Zambia 2020 [29/03/2022]. Available from: https://www.unaids.org/sites/default/files/country/documents/ZMB_2020_countryreport.pdf.
4. Republic of Zambia. National Tuberculosis and Leprosy Programme Tuberculosis Manual: Republic of Zambia; 2017 [12/02/2021]. Available from: https://www.afro.who.int/sites/default/files/2019-06/4%20%20Tuberculosis%20Manual%20for%20Zambia_Final.pdf.
5. National AIDS Commision Malawi. 2021 HIV Epidemiological Estimates for Malawi 2021 [29/03/2022]. Available from: http://www.aidsmalawi.org.mw/view-resource/2021%20HIV%20EPIDEMIOLOGICAL%20ESTIMATES%20FOR%20MALAWI.pdf.
6. USAID. TB CARE II in Malawi 2020 [30/03/2022]. Available from: https://www.urc-chs.com/wp-content/uploads/urc-tbcare-malawi-2020.pdf.
7. The Global Tuberculosis Community Advisory Board. Activists call on countries and donors to immediately scale up use of life-saving TB LAM test: TB Online; 2018 [02/12/2021]. Available from: https://www.tbonline.info/media/uploads/documents/lam_demo_press_release_union2018_v4_10.23.18_endorsements_final.pdf.
8. Singhroy DN, MacLean E, Kohli M, Lessem E, Branigan D, England K, et al. Adoption and uptake of the lateral flow urine LAM test in countries with high tuberculosis and HIV/AIDS burden: current landscape and barriers. Gates Open Res. 2020;4:24. Epub 2020/04/16. doi: 10.12688/gatesopenres.13112.2. PubMed PMID: 32185366; PubMed Central PMCID: PMCPMC7059561.
9. Ardizzoni E, Fajardo E, Saranchuk P, Casenghi M, Page AL, Varaine F, et al. Implementing the Xpert(R) MTB/RIF Diagnostic Test for Tuberculosis and Rifampicin Resistance: Outcomes and Lessons Learned in 18 Countries. PLoS One. 2015;10(12):e0144656. Epub 2015/12/17. doi: 10.1371/journal.pone.0144656. PubMed PMID: 26670929; PubMed Central PMCID: PMCPMC4682866.
10. Albert H, Nathavitharana RR, Isaacs C, Pai M, Denkinger CM, Boehme CC. Development, roll-out and impact of Xpert MTB/RIF for tuberculosis: what lessons have we learnt and how can we do better? European Respiratory Journal. 2016;48(2):516-25. doi: 10.1183/13993003.00543-2016.
11. Malawi Ministry of Health and Population. Malawi Guidelines for Clinical Management of HIV in Children and Adults 2018 [17/12/2021]. Available from: https://differentiatedservicedelivery.org/Portals/0/adam/Content/yb4xSSLvE0SW98_z7wTm_w/File/Malawi%20Clinical%20HIV%20Guidelines%202018%20(1).pdf.
12. Malawi Go. National Tuberculosis Control Programme Manual. In: Population MoHa, editor.: Government of Malawi; 2017.

13. Fazilla Tembo. Gene Xpert a vital tool in detecting tuberculosis: Nyasa Times; 2021 [29/03/2022]. Available from: https://www.nyasatimes.com/gene-xpert-a-vital-tool-in-detecting-tuberculosis/.
14. USAID. MALAWI REPUBLIC TUBERCULOSIS ROADMAP OVERVIEW, FISCAL YEAR 2021 2021 [30/03/2022]. Available from: https://www-origin.usaid.gov/sites/default/files/documents/Malawi_TBRM21_TB_DIAH_version_Final.pdf.
15. Republic of Zambia. Zambia Consolidated Guidelines for Treatment and Prevention of HIV Infection: Republic of Zambia; 2020 [12/02/2021]. Available from: https://www.moh.gov.zm/wp-content/uploads/filebase/Zambia-Consolidated-Guidelines-for-Treatment-and-Prevention-of-HIV-Infection-2020.pdf.
16. USAID. Strengthening the GeneXpert Network : bringing rapid TB testing to all in Zambia: Challenge TB; 2019 [30/03/2022]. Available from: https://www.challengetb.org/publications/tools/briefs/Zambia_GeneXpert_Technical_Brief.pdf.
