# Supplementary material for: User perspectives and preferences on a novel TB LAM diagnostic (Fujifilm SILVAMP TB LAM)–a qualitative study in Malawi and Zambia
Source: PLOS Glob Public Health. 2022 Jul 19;2(7):e0000672. doi: 10.1371/journal.pgph.0000672 (PMC10021253; doi:10.1371/journal.pgph.0000672)
Supplement: S2 File — Final codebook developed after saturation of interviews was reached considering the interview transcripts, topic guides and field notes. This codebook was applied to the entire data set before establishing descriptive memos and themes. (PDF) [file pgph.0000672.s002.pdf]

Supplement “User perspectives and preferences on a novel TB LAM diagnostic (Fujifilm SILVAMP TB LAM) – a qualitative study in Malawi and Zambia”

S2 File CODEBOOK**:** Bullet points are our codes, organised under different headings

BACKGROUND/ HISTORY PARTICIPANT

- Interviewee information: introduction of patient/ HCP, who they are, stating daily routine, work history
- Discovering HIV: when were they diagnosed, what symptoms did they have, why did they go to the doctor
- Reasons for TB testing: why did they get tested, what symptoms did they have? Ignoring symptoms and not getting tested.
- TB detection difficulties ARVs: patients on ARVs making it hard to detect TB due to lower viral load
- Concluding words: end of the interview, no added value to the comments said

DISEASE AWARENESS (OR HANDLING)

- Patient Agency: patients disagreeing with course of treatment, insisting on further check-up if symptoms persist, questioning of testing method, patient preference for a specific method
- Accepting testing and diagnosis: accepting outcome, reaction to a positive or negative diagnosis, understanding the need of being tested
- Confidentiality: any time someone expresses any thoughts on how they want to hide the fact that they are getting tested, or if someone doesn’t care about sharing their testing and discretion, stigma surrounding testing
- Cost of testing to patients: how much does testing cost for the patients
- Accessing healthcare facilities: distance travelled, difficulty reaching facilities, budget for transportation
- Waiting on HPC: having to wait at the hospital for an appointment/ to see a HCP, including waiting in a queue
- Trust in doctors: whenever someone expresses opinions such as “it is up to the doctor to decide” etc.

SAMPLES

- Instructions for sample collection: what were the patients told/ given, when were they told to bring in the samples; What is the HCP required to collect and in what quantities
- Sputum sample
- Blood sample
- Urine sample

- Sanitary facilities: facilities used/ needed for sample collection
- Stool sample
- Trust in samples: generally what samples are most trusted by users and why
- Sample transportation: if a sample has to be brought to an external laboratory f.i.

TEST PERFORMANCE

- Diagnostic yield: sensitivity of tests
- False results: what happens with a FP and FN result
- Confidence in test and diagnosis: a low sensitivity or inability to correctly diagnose TB makes patient lose confidence of the test, patient perception on testing methods
- Combining testing methods: combination of testing methods to diagnose, expressing more trust in using a combination of testing methods or factors to ensure that the disease is diagnosed correctly
- Testing algorithm: decision makers giving information on current use of which tests and why (guidelines, health ministry etc.)
- Test distribution: within the countries, what tests are available and where?
- Turnaround time: how long did it take to get the results after giving sample? Opinions on ideal TAT, worrying while waiting for results, impact of faster treatment on the patient
- Balancing accuracy vs time: mentioning that it doesn't matter how long the test results take to come out, and prefer the correct diagnosis over this
- Result interpretation: How easy or difficult is it to interpret the result read outs?
- Testing capacity: how many tests can be conducted per day

POST-TESTING

- Follow-up: instructions given, opinions on FUs, why patients might miss FUs
- Post TB diagnosis: does the patient start treatment right after diagnosis? Where do they get treated?
- Treatment side effects

TEST SPECIFIC

- Alere-specific
- CD4-specific
- Xpert-specific
- Microscopy specific
- Culture specific
- X-ray specific
- Cost of tests

FUJILAM SPECIFIC

- Deployment FujiLAM: challenges with implementation, importance of implementation at small facilities, possibility of using alongside CD4 tests, raising awareness for the test, personnel needed
- Handling of FujiLAM: related comments to the usage of the FujiLAM test including the kit and packaging
- Introduction to FujiLAM: when did you first hear of FujiLAM? First impressions
- Study-specific information: any information that specifically in related to the study

SUBGROUPS

- TB in children: everything related to children, will merge this information with Malawi when we get more information about children
- Inpatient vs. outpatient: put any codes that differentiate between in- and outpatients
- **REST**: code that doesn't make sense or doesn't contain anything that could be useful
