# Supplementary material for: User perspectives and preferences on a novel TB LAM diagnostic (Fujifilm SILVAMP TB LAM)–a qualitative study in Malawi and Zambia
Source: PLOS Glob Public Health. 2022 Jul 19;2(7):e0000672. doi: 10.1371/journal.pgph.0000672 (PMC10021253; doi:10.1371/journal.pgph.0000672)
Supplement: Attachment — Submitted filename: Response to reviewers_R1.docx [file pgph.0000672.s004.docx]

| **Reviewer 1 Comments to the Author** | **Response from Author** |
| --- | --- |
| Check grammar. A few errors were found where authors used the wrong words, or included words that weren't supposed to be in the sentence. E.g., Line 32, should read 'sub-Saharan Africa' not 'Sub-Saharan Africa'. | Thank you for this comment. We thoroughly checked the manuscript and made appropriate changes throughout the document. |
| Aim for clarity in writing. Also, beware of ethics inferred through writing. E.g., Line 11, 'decision makers' is never explained. Who are DMs? What role(s) do they play in the TB programme? Or, the HIV programme?  Lines 28, 193, 'respondents' removes agency from people with whom we conduct research, 'participants' is a preferable term. | Thank you for pointing out these details. We have added a description of DMs in the abstract (line 10-11) and in the methods (lines 169-173). We want to mention that a broad description of DM is given in order to preserve the confidentiality of our study participants, since people in certain roles (e.g., directors or heads of a program at a national level) are few and relatively easy to identify. For this reason, we have tried to balance the reviewer’s request with our duty to ensure the ethical aspects of the study.  Regarding the use of the word ‘respondent’, we have incorporated the changes suggested by the reviewer across the manuscript. |
| Lines 44-46, 'Abbott’s Alere Determine TB-LAM (USA; henceforth called AlereLAM), has shown to reduce mortality from TB disease in patients with advanced HIV despite a limited sensitivity.' The onus is placed on the reader to determine that the LAM test resulted in earlier TB diagnoses, earlier TB diagnoses resulted in earlier treatment initiation and earlier treatment initiation results in reduced mortality. | We appreciate this suggestion and have rephrased the sentence, as well as briefly described the cascade to facilitate reading (line 115). |
| Line 361, 'patients and children'. Are children not also patients? Similarly in Line 364, 'children and people'. Are children not people? | We have edited the sentences to minimize the potential confusion they may have generate (lines 766-767; 771). |
| Existing section entitled 'context,' provides very little context to the burden of TB and HIV in Malawi and Zambia. This 'epidemiological' context is important to show the reader why FujiLAM could help change NTPs in similar contexts. In addition to your description of use of LAM testing, it is important to include information about the public health system: do TB patients generally receive care at public hospitals? In other southern African countries, TB patients receive care at primary health care facilities. However, sputum and Xpert tests are not done at these facilities and samples are sent to other health facilities which have the technologies with which to test samples. Without this information it is difficult to determine if, for example, Lines 129-131, are true. | We appreciate the reviewer’s remark. We added more details and moved the “context” section into a separate supplement file, allowing us to provide a more comprehensive overview of the context. |
| Existing section entitled 'FujiLAM test characteristics and use instructions,' could be summarised elsewhere. | This section is an important aspect of our methodology as it describes the core device and innovation of our study. Nonetheless, the position of the section was changed hoping that it allows a better reading flow of the section. |
| Table 1 could be better presented. | We modified the table and streamlined the presentation of the data to facilitate reading. |
| 'Data entry and analysis' is confusing. The description of data analysis approach (Lines 156-164) aligns well with File S1. However, then findings are 'presented following the health equity implementation framework' (Line 165). This is not methodologically sound. The description of methods used aligns closely with an inductive thematic analysis approach, whereas the use of the 'equity implementation framework' aligns with a deductive thematic analysis approach. The former allows the data, through iterative analysis, to determine themes. The latter presumes themes included in the equity implementation framework are appropriate to describe the data. Additionally, the sub-themes used in the results section are not introduced nor do they align with File S1 or the 'equity implementation framework.' Consider using the themes arrived at in File S1.  If the authors would like to use the equity implementation framework, then the onus is on them to describe the framework, and illustrate its applicability to the presented study. This is not done. The only mention of the health equity implementation framework is in Line 70, where its appropriateness is presumed. | We have edited the ‘Data entry and analysis’ section to further elaborate on our methodological approach, the Health Equity Implementation Framework (HEIF) and its relevance to the study (lines 333-365). In summary, similar to the analysis approach outlined by Pope et al^1^, the HEIF was selected to structure the themes along determinants of effective implementation. Thus, we used the framework to contextualize our result and organize them, rather than solely applying a deductive thematic analysis approach.  We expect that this addition to the methods allows the reader to have a better understanding of our approach to data analysis.  ^1^ Pope, C., Ziebland, S., & Mays, N. (2000). Qualitative research in health care. Analysing qualitative data. BMJ (Clinical research ed.), 320(7227), 114–116. https://doi.org/10.1136/bmj.320.7227.114 |
| There are 42 interviews, 20 of which are among patients. However, patients views (and quotes) are rarely described in the results. This is strange given the authors argue FujiLAM will improve health equity. Consider being more purposeful in presenting both patients and DMs/HCPs perspectives throughout the results, like you do for the 'recipient factors' theme. | We appreciate the reviewer’s feedback. Patient perceptions and voices (e.g., quotes) were added to the respective themes for a more balanced presentation of participants. However, since the interview guides were tailored for each group of participants some question covered in the innovation and context factors were not included in the patient’s interviews. Therefore, we were not able to include patients’ perceptions or quotes on themes emerging from the interviews of the professional participants. |
| Given the disconnect between the methodological approach and the application of the 'health equity implementation framework,' as well as the lack of explanation of the frameworks' application to the data, the results are difficult to follow. E.g., the existing section, 'Criteria for CD4 count and its potential impact on FujiLAM’s use and implementation' is more about changes in guidelines than the innovation itself. It is unclear how/why it belongs in the 'Innovation factors' theme. | We appreciate the reviewer’s remark. Hopefully, the answer prior provides a comprehensible explanation of our methodological use of the framework.  We strongly believe that guidelines and instructions play a vital role in the test’s qualitative evaluation. Nonetheless, we acknowledge that this section would be better placed below “context factors” (lines 831-871). |
| A strong case is made of the potential of FujiLAM to improve existing health systems practices and policies for NTPs. A good overview of results is provided.  Limitations are adequately noted. However, Lines 540-544, do not adequately acknowledge that 'urban public hospitals' are not the primary facilities in which patients seek TB and HIV care. This should also have been included in the description of the 'context'. | Thank you for your comment.  The public urban hospitals where our study took place offered TB and HIV care to all patients. We acknowledge that that first-line treatment is also offered at primary health care facilities and have added a sentence to the limitations in (lines 1024-1027) and to the context (S1 File). |
| This study cannot adequately establish whether FujiLAM can be decentralised to rural clinics, and taken up as part of the diagnostic processes in these clinics, because the data are not from this setting. At most, they can claim HCPs, DMs and patients at two urban public hospitals suggest FujiLAM can and should be decentralised for use in primary health care facilities in Zambia and Malawi. | Thank you for your remark. We acknowledged this as a limitation of our study and expanded this explanation in the discussion (lines 1024-1027). |

| **Reviewer 2 Comments to the Author** | **Response from Author** |
| --- | --- |
| 1. The script is generally well written | Thank you, we appreciate the reviewer’s comment. |
| 2. There are few spelling errors in the text, e.g. Below in line 96 has been spelled as "bellow", i encourage the authors to check for spelling errors. | We appreciate the reviewer’s remark and have thoroughly checked the manuscript for further errors. |
| 3. The methodology contains material relevant in the background, especially the items stated from line 81 to 117, methodology will flow well if these are moved to the background. | Thank you for this comment. We have removed the ‘context’ (former lines 81-117) from the methods section and have added a supplement file (S1). The S1 file provides further information of the current context in Zambia and Malawi, in accordance with Reviewer one, who suggested a more comprehensive overview. To allow a better flow of the methods section, we then decided it would be best to add the context overview in a supplement file. |
| 4. In the discussion, the authors have stated the role of the Urine LAM both Fuji and Alere, however, they did not indicate that all LAM positive patients require further evaluation with the Genexpert, especially that WHO classifies Zambia as one of the high MDR TB burden countries, this is useful in ruling or diagnosing resistance to rifampicin. | We appreciate the reviewer’s comment and have acknowledged this. A sentence was added to the discussion shedding light onto this limitation of LAM tests (lines: 971-974). |
| 5. The 1st sentence in the conclusion is not clear, I could not appreciate the what the authors meant by facilitators. Did they mean factors? | We appreciate the feedback and want to address the concern. With facilitators used the definition by Bach-Mortensen et al.^1^: as “the factors that enable the implementation of evidence-based interventions”. We have briefly added the definition to the text for clarity (lines 349-350).  ^1^ Bach-Mortensen, A.M., Lange, B.C.L. & Montgomery, P. Barriers and facilitators to implementing evidence-based interventions among third sector organisations: a systematic review. Implementation Sci 13, 103 (2018). <https://doi.org/10.1186/s13012-018-0789-7> |
| 6. The reviewers may wish to review the diagnostic algorithm for Zambia, it indicates that LAM can be used in Patients with Sepsis, this is missing in their background statement about the use of LAM in Zambia. | We appreciate that the reviewer pointed this out. The missing information has been included in the background section (line 133) while more details are included in the S1 file. We consulted the Zambian guidelines of 2017 and 2020 and could identify the term “severely ill” among eligibility criteria for LAM test. We hope that our revised phrasing is appropriate. |
